# High resolution mapping and candidate gene identification of downy mildew race 16 resistance in spinach

**DOI:** 10.1186/s12864-021-07788-8

**Published:** 2021-06-26

**Authors:** Gehendra Bhattarai, Wei Yang, Ainong Shi, Chunda Feng, Braham Dhillon, James C. Correll, Beiquan Mou

**Affiliations:** 1 Department of Horticulture, University of Arkansas, Fayetteville, AR 72701 USA; 2 Department of Entomology and Plant Pathology, University of Arkansas, Fayetteville, AR 72701 USA; 3 Department of Plant Pathology, University of Florida - Fort Lauderdale Research and Education Center, Davie, FL 33314 USA; 4 USDA-ARS Crop Improvement and Protection Research Unit, Salinas, CA 93906 USA

**Keywords:** Spinach, Downy mildew, Disease resistance, GWAS, Mapping, Breeding

## Abstract

**Background:**

Downy mildew, the most devastating disease of spinach (*Spinacia oleracea* L.), is caused by the oomycete *Peronospora effusa* [=*P. farinosa* f. sp. *spinaciae*]. The *P. effusa* shows race specificities to the resistant host and comprises 19 reported races and many novel isolates. Sixteen new *P. effusa* races were identified during the past three decades, and the new pathogen races are continually overcoming the genetic resistances used in commercial cultivars. A spinach breeding population derived from the cross between cultivars Whale and Lazio was inoculated with *P. effusa* race 16 in an environment-controlled facility; disease response was recorded and genotyped using genotyping by sequencing (GBS). The main objective of this study was to identify resistance-associated single nucleotide polymorphism (SNP) markers from the cultivar Whale against the *P. effusa* race 16.

**Results:**

Association analysis conducted using GBS markers identified six significant SNPs (S3_658,306, S3_692697, S3_1050601, S3_1227787, S3_1227802, S3_1231197). The downy mildew resistance locus from cultivar Whale was mapped to a 0.57 Mb region on chromosome 3, including four disease resistance candidate genes (Spo12736, Spo12784, Spo12908, and Spo12821) within 2.69–11.28 Kb of the peak SNP.

**Conclusions:**

Genomewide association analysis approach was used to map the *P. effusa* race 16 resistance loci and identify associated SNP markers and the candidate genes. The results from this study could be valuable in understanding the genetic basis of downy mildew resistance, and the SNP marker will be useful in spinach breeding to select resistant lines.

## Background

Spinach (*Spinacia oleracea* L.) is a diploid (2n = 2x = 12), a majorly dioecious, wind-pollinated crop, and it is highly heterozygous. The majority of fresh market spinach (80% above) production is in California and Arizona, with more limited production areas in other states in the United States (US) [1, 2]. Spinach is highly nutritious with a high amount of vitamins, proteins, minerals, flavonoids and contains low calories [3, 4]. Spinach leaves are sold fresh, frozen, and in cans, and there is an increasing demand for spinach in the US and elsewhere. Fresh-cut spinach is used for salads, cooked singly or mixed with other vegetables, and added in soup, pizza, pasta, and many other dishes.

Downy mildew (DM), caused by the obligate oomycete *Peronospora effusa,* formerly known as *P. farinosa* f. sp. *spinaciae* (*Pfs*), remains the most economically important spinach disease. A total of 19 unique races of *P. effusa* have been documented [5–9], and 16 were reported in the last three decades. In addition to named races, there have also been a number of isolates with novel virulence patterns on a standardized set of differential germplasm. The downy mildew pathogens are highly host-specific for their life-cycle completion, have a short latent period of 6–8 days, and reproduce asexually by producing sporangia. Cool and humid weather favors germination and growth of downy mildew spores [10, 11]. A standardized set of differential cultivars is used to differentiate *P. effusa* races. The International Working Group on *Peronospora* (IWGP) coordinates the efforts to denominate new races for isolates showing novel and consistent virulences among the participating *P. effusa* race-typing labs [12]. The continuous emergence of new *P. effusa* races is a severe threat to the spinach industry. Significant increase in the production area in last two decades, planting in a higher density, year-round production, and planting of resistant cultivars with narrow genetic background increases selection pressure, continuous increase in organic production area provides a niche for *P. effusa* growth and multiplication, and these phenomena in combination are conducive for the emergence of a new race. New races are likely a result of asexual variation [13] and sexual recombination [13, 14] within the pathogen populations.

Although there are a number of disease management strategies for downy mildew of spinach, disease resistance is the most effective, economical, and environment-friendly disease management method [15–17]. More than 40% of spinach production in the US is organically grown, and for organic spinach production, genetic resistance is the only disease management option. For this reason, breeding for resistance to downy mildew is the primary objective of all spinach breeding programs [18]. Identifying additional resistance sources against *P. effusa* races is necessary to breed durably resistant cultivars to sustain commercial spinach production.

Downy mildew resistant spinach cultivars are bred using major gene resistance to a particular race of *P. effusa*, and these dominant resistant genes are known as *RPF* (Resistance to *Peronospora farinosa*) genes. Six different *RPF* loci were hypothesized to govern resistance against the *P. effusa* races [15], and so far, *RPF*1, *RPF*2, and *RPF*3 have been genetically characterized [19]. Commercial cultivars are developed using a single or in a combination of a few *RPF* genes. However, with the limited availability of genetic-genomic resources in spinach, little is known at the molecular level regarding the resistance genes against the downy mildew pathogen. Genetic investigation of the resistance sources and identifying molecular markers linked to the resistant genes are expected to facilitate R-gene pyramiding, breeding, and selection for new resistant cultivars.

The *RPF*1 locus governed by a single dominant allele falls on chromosome 3. A co-dominant marker, DM1, is 1.7 cM proximal from the *RPF*1 locus and has been widely used to select resistance alleles [20]. Similarly, marker 5B14r identified from the resistance gene analogs (RGA) linked to the *RPF*1 locus cosegregates with the DM1 marker [21]. Downy mildew resistance loci *RPF*1, *RPF*2, and *RPF*3 were mapped to a 1.5 Mb region of chromosome 3, and closely linked PCR markers that can distinguish the *RPF*1–3 loci were reported [19]. Mapping the DM1 marker in the spinach genome identified five nearby genes with the NBS-LRR domain and were predicted as candidate genes providing resistance to downy mildew in spinach [22]. The locus was further narrowed to a 0.89 Mb region, extending from 0.34 to 1.23 Mb, containing 14 putative disease resistance genes of which Spo12729, Spo12784, and Spo12903 were reported as the most likely candidate genes based on protein homology search between resistant and susceptible lines [23]. The *RPF* resistance locus was recently mapped between 0.39–1.23 Mb based on single SNPs and haplotype association analysis performed in multi-parent progenies [24]. Furthermore, the study of Bhattarai et al. [24] reported Spo12784, Spo12903, Spo12905, and Spo12821 as the candidate genes based on their close physical position (1–18 Kb) from the associated SNPs. However, the candidate genes reported in all previous studies were predicted from the Sp75 reference assembly [22] that does not contain known downy mildew resistance genes. Thus the effective gene in the resistance line may not have orthologs in the reference genome of the susceptible cultivar.

Genetic linkage mapping and genomewide association mapping are commonly used to identify linked markers and associated genomic regions controlling the phenotype of interest. The bi-parental QTL mapping requires the development of progeny population segregating for a trait of interest, while association mapping can be conducted in diverse germplasm or mixed populations. Association analysis allows mapping the trait and discovers candidate genes governed by qualitative and quantitative traits in any population showing variation for the trait of interest, as conducted in spinach [24–27]. Association mapping analysis has been reported using the F2 population in plants and animals [28–32], including the mapping of the downy mildew resistance in hops (*Humulus lupulus* L.) [33] to identify genetic architecture and genomic region and candidate genes governing the economically important trait.

A genetic linkage map of spinach was constructed for a backcross population segregating for gender (male, female) while the markers were grouped into seven linkage groups, although spinach has only six chromosomes [34]. The male and female sex segregated in a 1:1 ratio, and the sex determination locus was mapped to a single locus. Another SNP based genetic linkage map was constructed in spinach using F_2:3_ population segregating for nitrogen use efficiency (NUE) [35]. SNPs were identified by sequencing parental transcriptome, and the segregating population was genotyped by converting homozygous SNPs polymorphic in between two parents into Kompetitive allele specific PCR (KASP) assay. The genetic map was constructed using 283 SNP markers that were grouped into six linkage groups and QTLs associated with the NUE in spinach were detected. In another study, Specific-locus Amplified Fragment Sequencing (SLAF-seq) based markers were used to construct a genetic map and map spinach sex locus [36].

The hybrid spinach cultivar Whale contains downy mildew resistance locus *RPF*3, while the cultivar Lazio contains the *RPF*2 and *RPF*4 loci, and their resistance responses to the *P. effusa* races are known [5, 6]. Spinach cultivar Whale is resistant to *P. effusa* races 1–3, 5, 8–9, 11–12, 14, 16, and susceptible to *P. effusa* races 4, 6–7, 10, 13, 15. Similarly, Lazio is resistant to races 1–10, 15, and is susceptible to races 11–14, 16. As the cultivar Whale is resistant to *P. effusa* race 16 and the Lazio is susceptible, this study aimed to map the *RPF*3 locus and identify resistance using the progeny population of cultivars Whale and Lazio. This mapping effort provides an increased resolution of the resistance region and identifies SNP markers and the candidate genes associated with the downy mildew resistance from cultivar Whale.

## Results

### Downy mildew response

Spinach cultivar Whale is resistant to *P. effusa* race 16, while Lazio is susceptible [5, 6]. A segregating population generated from a cross between the two cultivars, Whale and Lazio, were screened for resistance against race 16 of *P. effusa*. Expected downy mildew signs and symptoms were observed in the susceptible cultivar Viroflay and Lazio and the resistant cultivar Whale following *P. effusa* race16 inoculation. The spinach population showed downy mildew severity in a range of 0–100%, but disease response showed bimodal distribution as per the qualitative evaluation for the presence and absence of pathogen growth (data not shown). Qualitative disease incidence data scored for each line were used as phenotype data, and association analysis was conducted using the binary disease scores of 172 spinach lines (123 resistant and 49 susceptible) that remained after filtering for the individual lines with high missing SNP calls and parental lines.

### Genotyping-by-sequencing and SNP calling

**Fig. 1:**
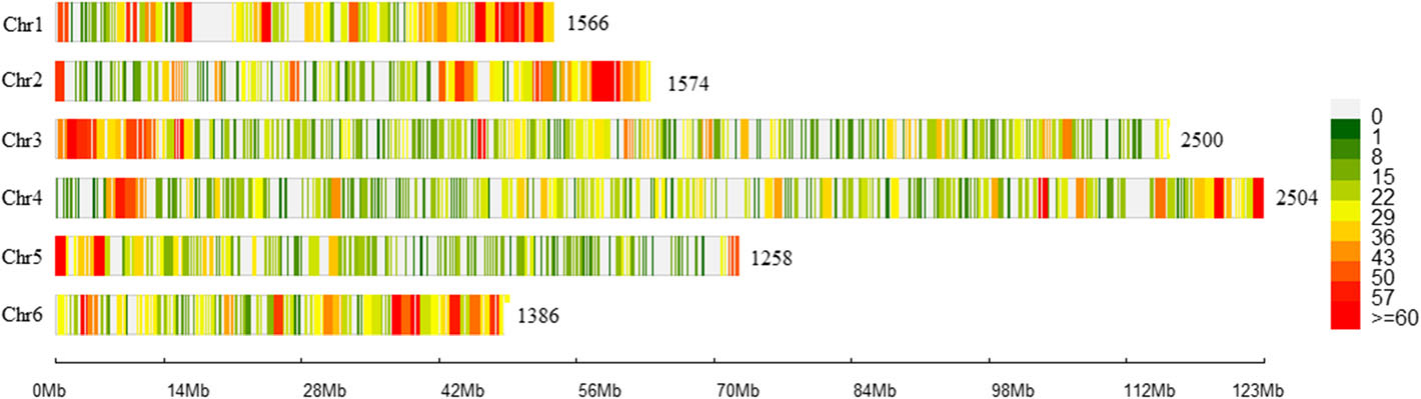
Genomewide distribution of the single nucleotide polymorphism (SNP) marker on six spinach chromosomes. The vertical axis shows chromosomes, the horizontal axis shows chromosome length in Mb, and the color represents the SNP density, the number of SNPs per window

Two hundred sixty-nine million raw reads were generated from the Illumina NovaSeq run, with an average of 1.38 million reads per sample. Sequencing adapters and low-quality bases were filtered and 263 million (98%) good reads were retained and aligned with the six chromosome sequences of the inbred spinach line Sp75 (http://www.spianchbase.org) [22, 37]. Fifty-one thousand SNPs were called using the TASSEL v2 pipeline, and the SNPs were named with the ‘Chr_position’ format as ‘S3_658306’. VCFtools filtered 43 k biallelic SNPs were imported in PLINK and filtered for missing calls (> 25%), individuals with more than 25% SNPs, MAF (< 2%), and HWE (> 1e-07) removed. The final filtered dataset contained 10,788 SNPs, and the distribution of SNPs on the six chromosomes of spinach was presented as a density plot (Fig. 1).

### Population structure

**Fig. 2:**
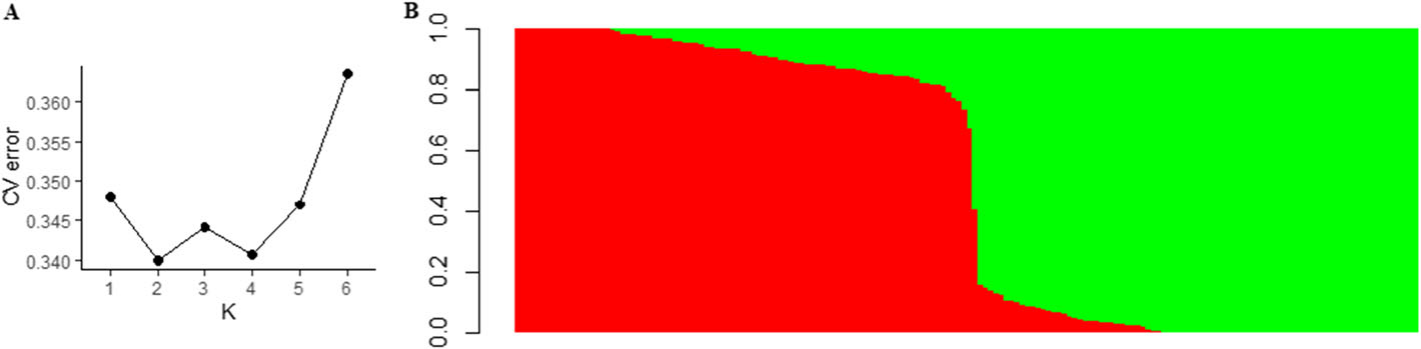
Population structure of the spinach population in this association panel. 2**A**. Optimum K was determined with the minimum cross-validation errors in the data for K. 2**B**. Grouping of genotypes in the association panel into two genetic sub-populations where the horizontal axis represents the spinach genotypes and the vertical axis represents the probability of genotypes belonging to different genetic groups. Spinach genotypes membership proportion to each population group are shown with a unique color, red (Q1) and green (Q2)

**Fig. 3:**
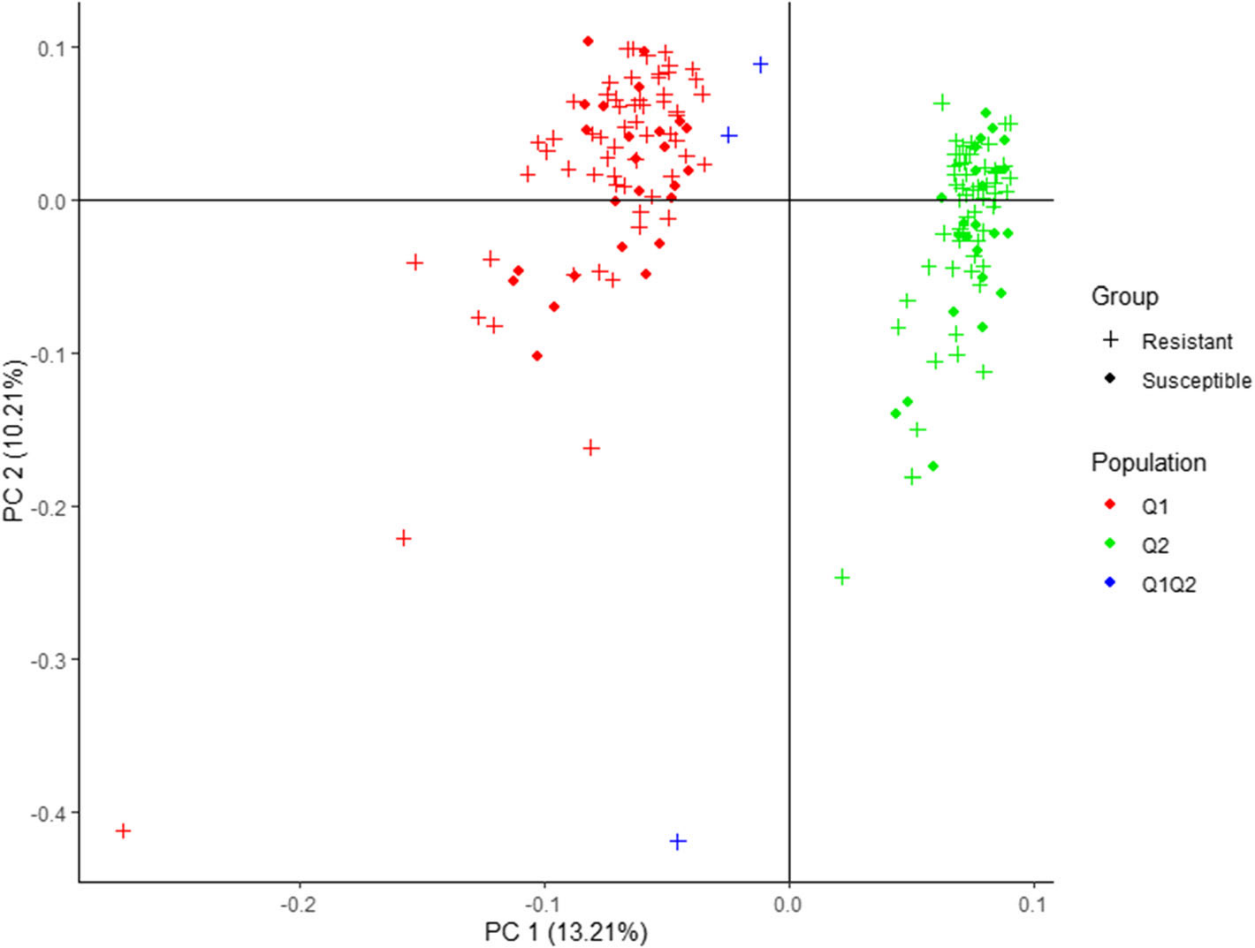
The principal component analysis (PCA) plot of the 172 spinach genotypes. The horizontal and vertical axis represents the first and second principal components, and the variances explained by each component are noted. Colors correspond to members of subpopulations Q1 (red), Q2 (green), and admixed group Q1Q2 (blue). Resistant and susceptible genotypes are resented by “plus” and “filled circle” signs

High correlated pairs of SNPs pruned in PLINK retained 7752 SNPs and were used to analyze the genetic structure on ADMIXTURE software. The ADMIXTURE cross-validation error supports two main clusters in the spinach panel (Fig. 2A). A membership cutoff of 0.75 was used to divide spinach genotypes into two sub-populations, and the genotypes with membership-coefficient < 0.75 were considered admixed (Fig. 2B). Of the 172 spinach genotypes, 85 were assigned to group 1; 84 were assigned to group 2; and the remaining three genotypes were assigned to an admixed group. Principal component analysis performed in PLINK showed the first two PC accounted for 13.21 and 10.21% of the total genetic variation, respectively. The first two PC differentiated the association panels into two genetic subgroups (Fig. 3), and a certain level of population structure was observed. Hence, the first two principal components on the IBS matrix of all pairs of individuals were used as covariates in PLINK to control population stratification. Similarly, PC covariates were computed in TASSEL and GENESIS to control the effect of population structure.

### Genomewide association analysis

Association analysis was conducted to identify genetic loci governing the resistance to race 16 of *P. effusa* in a panel of 172 spinach population. GWAS models were run using SNPs generated from GBS on multiple programs to determine consistent associations and avoid spurious associations. The Bonferroni significance threshold (LOD value > 5.34) was used to call for the association in all tested models.

**Fig. 4:**
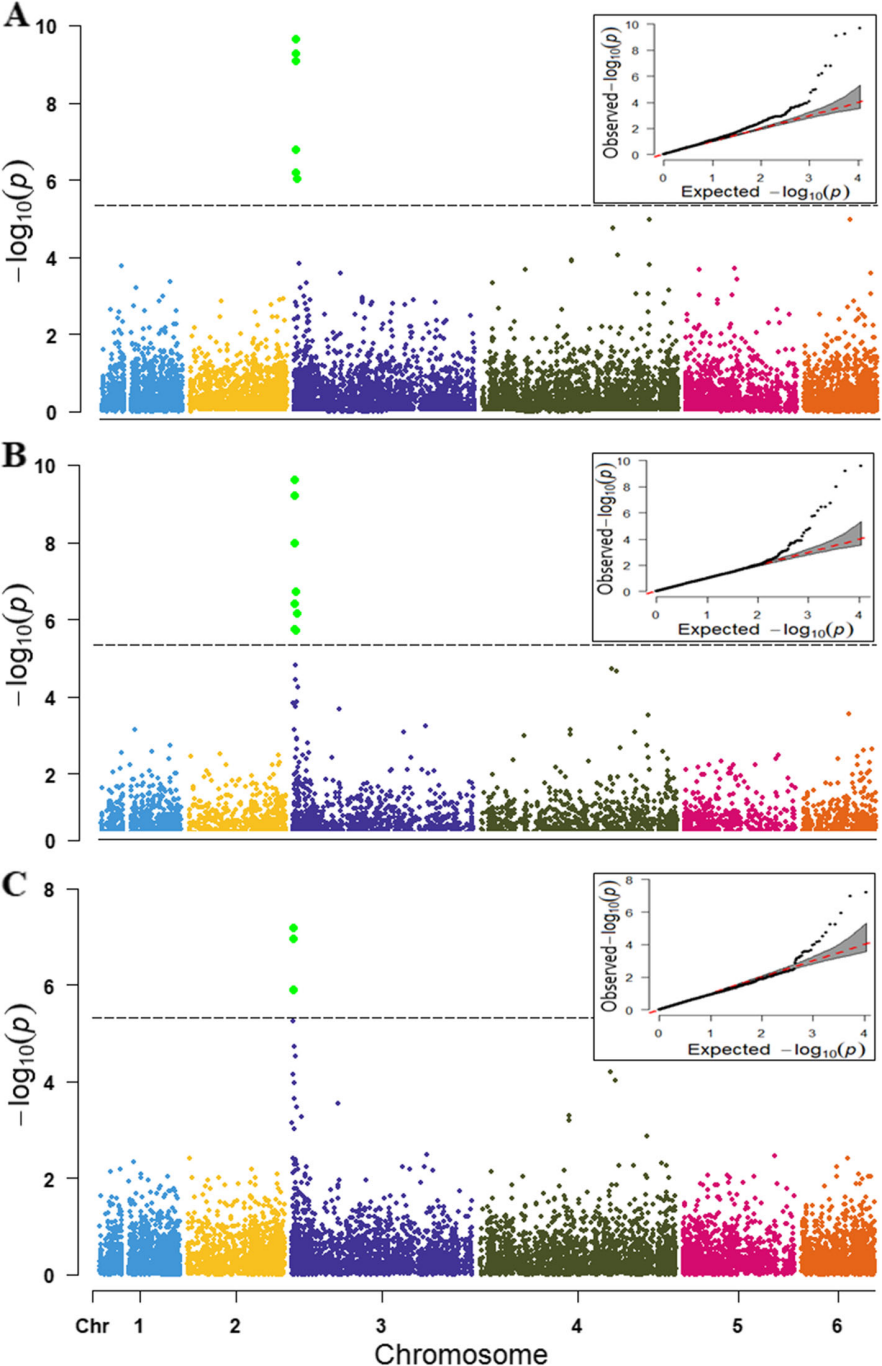
Manhattan and QQ-plots of genomewide associations of the *P. effusa* race 16 resistance in spinach using SMR (4A), GLM (4B) and MLM (4C) model in TASSEL. The horizontal axis in the plot represents the physical position of the SNP in the genome and the vertical axis shows the association power of each SNP with the trait expressed as -log_10_(*P*-value). The dashed line shows the Bonferroni-corrected genomewide threshold

In TASSEL, association analysis was performed using the SMR model, GLM model with the first two PC, and the MLM model by adding the PC and kinship matrices as covariates to control population structure and family relatedness. Seven and nine significant SNPs were detected in the SMR and GLM model (Table 1, Fig. 4A), while only three of the SNPs loci showed significance in the MLM model (Fig. 4B). All significant SNPs except S3_1476491were present in the 0.66–1.23 Mb (0.57 Mb) region of chromosome 3, which was only significant in the SMR model. The SNP loci S3_1050601, S3_1231197, and S3_692697 were detected on both the GLM and the MLM model in TASSEL. The phenotypic variance (R^2^) explained by these three SNPs loci averaged 20% in the GLM and MLM models.

**Table 1:** SNP markers significantly associated with *P. effusa* race 16 resistance in population segregating from cultivar Whale

SNP marker^a^	Alleles	Reference allele^b^	R allele	S allele	MAF	-log_10_P Value^c^						R^2^ (%) SMR^d^	Candidate gene ID^e^	Functional annotation	Distance in Kb from the gene
						TASSEL SMR	TASSEL GLM	TASSEL MLM	PLINK (GC)	GENESIS	T-test				
S3_658,306	G/A	G	A	G	0.47	6.20	5.76	4.15^f^	4.03^f^	5.47	6.32	18.5	Spo12736	NB-ARC; leucine-rich repeat (LRR)	8.92 upstream
S3_692697	A/T	A	T	A	0.32	9.66	7.98	5.92	4.49^f^	5.89	3.64^f^	24.1	Spo12784	NB-ARC; leucine-rich repeat (LRR)	2.69 downstream
S3_1050601	T/A	T	T	A	0.5	9.09	9.22	7.20	4.14^f^	5.51	5.71	22.1	Spo12908	CC-NBS-LRR disease resistance protein	10.83 downstream
S3_1227787	C/A	C	C	A	0.47	6.80	6.42	5.25^f^	4.01^f^	5.37	5.91	19.0	Spo12821	CC-NBS-LRR disease resistance protein	7.86 downstream
S3_1227802	G/A	G	G	A	0.47	6.80	6.42	5.25^f^	4.01^f^	5.37	5.91	19.0			7.88 downstream
S3_1231197	C/T	C	T	C	0.46	9.27	9.61	6.96	5.39	7.35	8.26	22.7			11.27 downstream

^a^ Position of SNP marker on respective chromosome in basepairs. The SNP marker S3_658,306 is located on chromosome 3 and positioned at 658306 bp

^b^ Alleles on the Sp75 reference genome [22]

^c^ Four different association models were performed on three different programs. The principal components (PC) were used in TASSEL general linear model, and the PC and kinship covariates were used in the TASSEL mixed linear model. PC was used to conduct the logistic regression in PLINK, and the genomic control (GC) statistic was reported. Mixed model analysis in GENESIS was run using inbuilt PC-AiR and kinship matrices

^d^ Phenotypic variance (%) explained by the marker from the TASSEL single marker regression model

^e^ Candidate genes within the associated region were searched in the SpinachBase database (http://spinachbase.org/)

^f^ The SNP association signals were below the Bonferroni threshold in this model. However, the association signals on other models support the SNP as of a high-confidence association, and the result was presented based on association reports obtained from multiple models

**Fig. 5:**
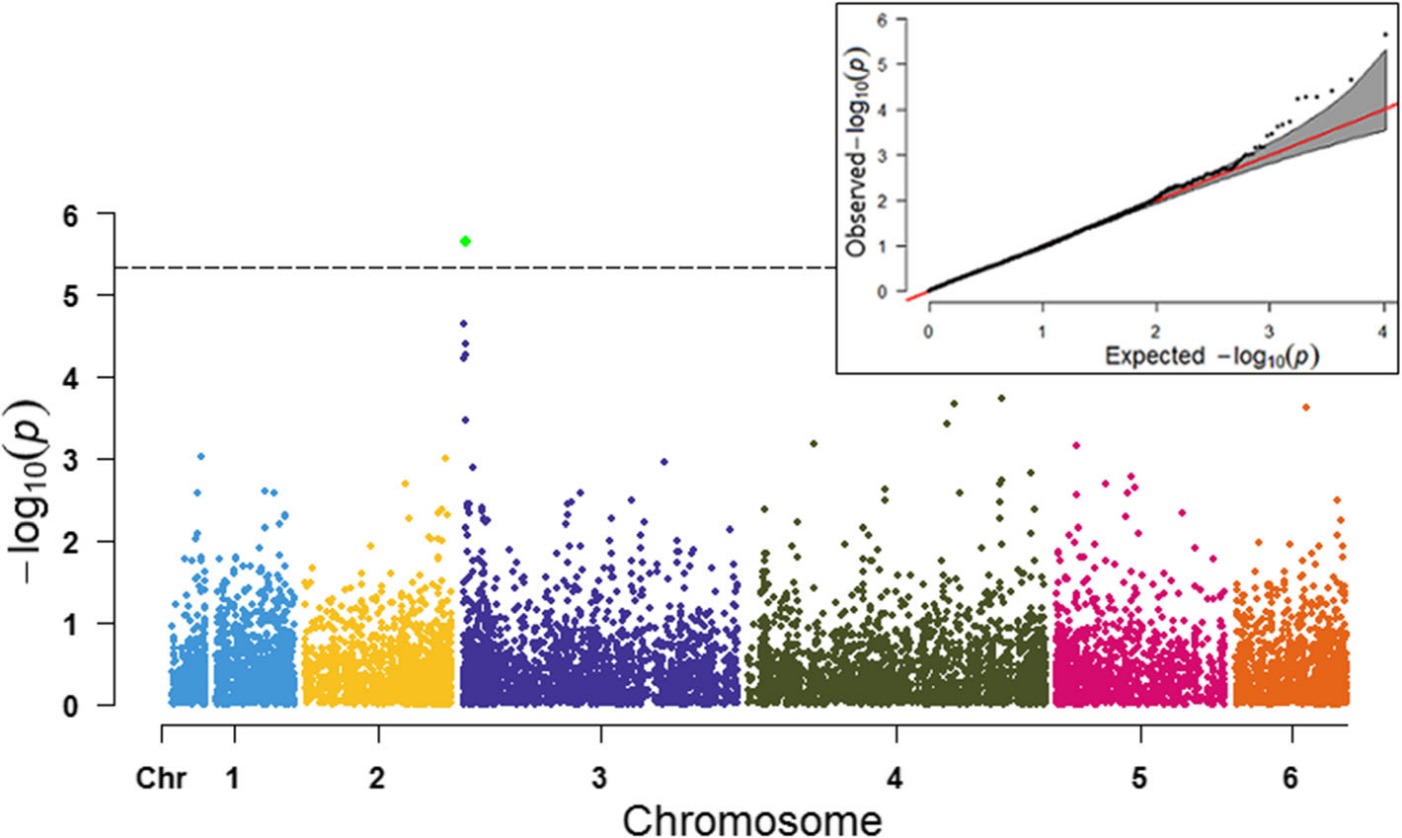
Manhattan and QQ-plots of genomewide associations of the *P. effusa* race 16 resistance in spinach using a logistic regression model including principal components in PLINK. The horizontal axis in the plot represents the physical position of the SNP in the genome and the vertical axis shows the association power of each SNP with the trait expressed as -log_10_(*P*-value). The dashed line shows the Bonferroni-corrected genomewide threshold

Next, a logistic regression model was run in PLINK using the first two PCA covariates to control population structure. PCA clustering was performed in PLINK using the LD pruned SNPs (7752) and the IBS values calculated from the LD pruned SNPs. Only one SNP locus (S3_1231197) exceeds the Bonferroni threshold in the PLINK logistic regression model (Fig. 5).

**Fig. 6:**
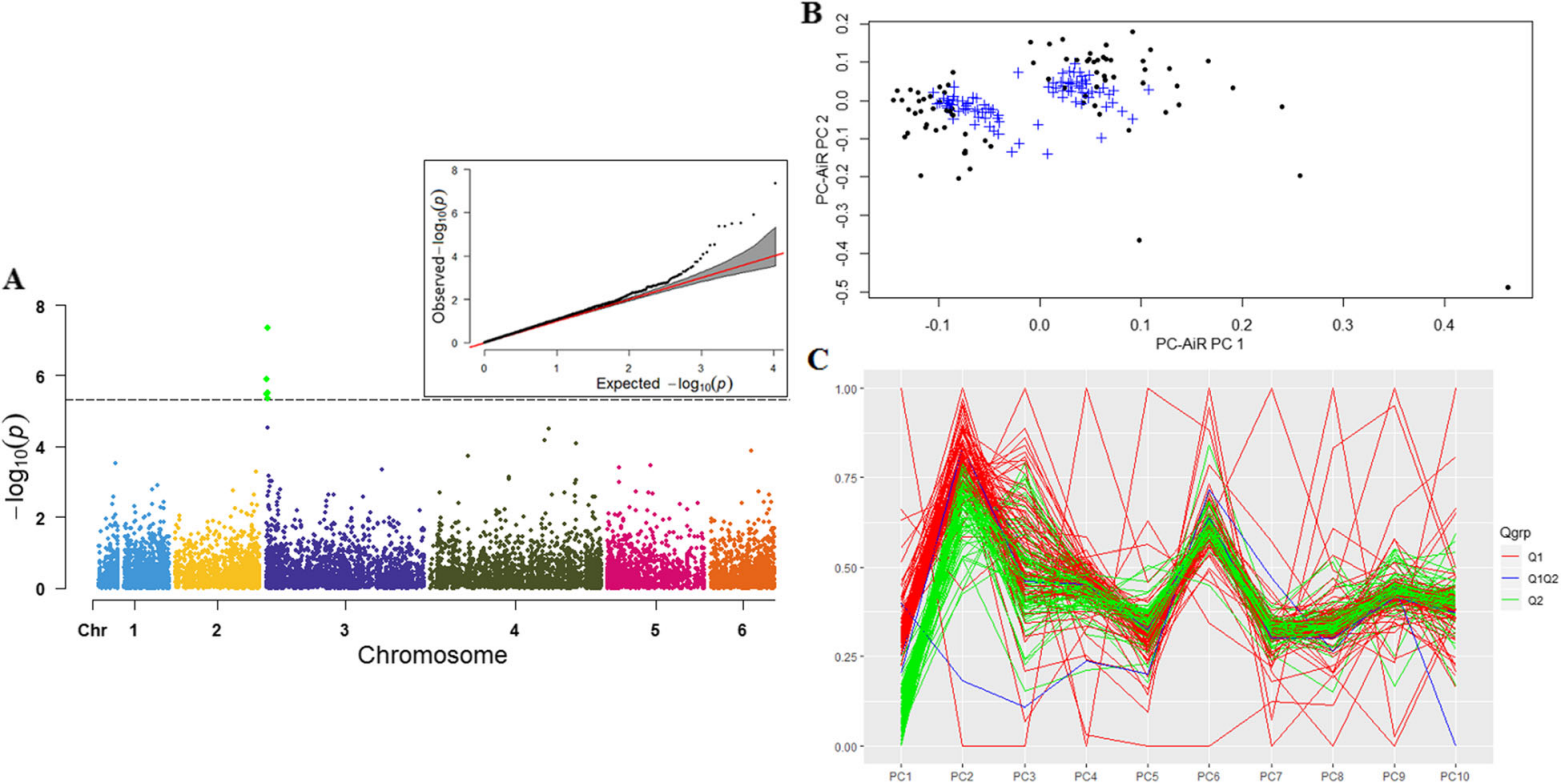
Manhattan and QQ-plots (6**A**) of genomewide associations of the *P. effusa* race 16 resistance in spinach using the GENESIS program. The horizontal axis in the plot represents the physical position of the SNP in the genome and the vertical axis shows the association power of each SNP with the trait expressed as -log_10_(P-value). The dashed line shows the Bonferroni-corrected genomewide threshold. The principal component analysis (6**B**) and parallel coordinates plot (6**C**) showed two PCs separate the samples and are colored according to the color code in the ADMIXTURE generated plot

Again, the logistic mixed model analysis was fitted in the GENESIS package in R using the genetic relatedness matrix estimated from PC-AiR and PC-Relate, and the Score test was used to assign the significance. The PC-AiR and parallel-coordinate plot showed two PCs informative of the ancestry in the samples, and the first two PCs separate the population (Fig. 6). Six SNPs (S3_1231197, S3_692697, S3_1050601, S3_658,306, S3_1227787, S3_1227802) were significantly associated with the *P. effusa* race 16 resistance in the GENESIS model (Fig. 6), and all these SNPs were commonly identified in the TASSEL GLM model.

The *t*-test analysis was performed on these six SNPs to confirm their allelic association with the phenotypic variation. The *t*-test analysis in this study showed a significant difference between two alleles across the resistant and susceptible panel at all six SNPs associated with *P. effusa* resistance in this panel, corroborating results obtained from previous GWAS models (Table 1). Significant SNPs commonly detected on multiple programs and association models were considered with high confidence to associate with the *P. effusa* race 16 resistance, and candidate genes within these six SNP regions were explored.

### Haplotype analysis and identification of candidate gene

**Fig. 7:**
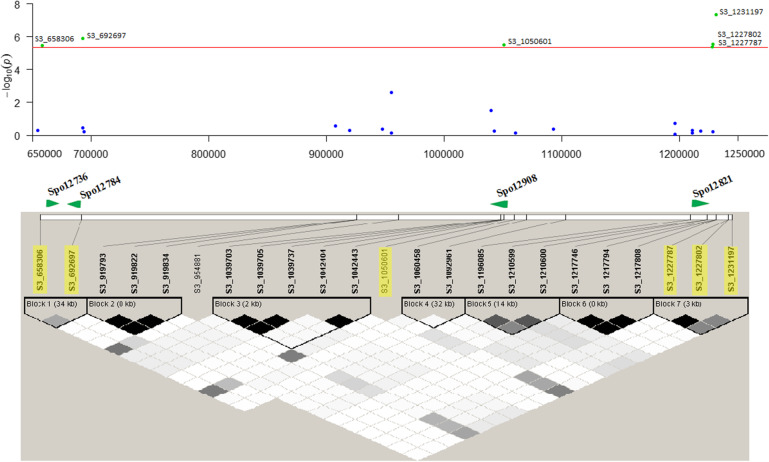
Regional association plot and candidate genes for the *P. effusa* race 16 resistance in spinach. The Manhattan plot of *P. effusa* association between 0.65 Mb to 1.25 Mb of chromosome 3. The horizontal and vertical axis represents the physical position of the SNP in the genome and the strength of association of each SNP with the trait expressed as -log_10_(P-value), and the red line shows the Bonferroni-corrected genomewide threshold. The middle panel shows the disease resistance candidate genes in the associated region and is highlighted in green and named in bold font. The lower panel shows linkage disequilibrium (LD) in the *RPF*3 associated region (0.65–1.23 Mb) based on pairwise r^2^ values. The gray color indicates the intensity of r^2^ (white for r^2^ = 0, shades of gray for 0 < r^2^ < 1, and black for r^2^ = 1

Haplotype block analysis was performed using 23 SNPs in the 0.57 Mb region of chromosome 3 associated with the *P. effusa* race 16 resistance. Seven haplotype blocks were observed in the region, with two blocks containing the five associated SNPs. SNP S3_658,306 and S3_692697 associated with *P. effusa* race 16 resistance were in a block with a moderate LD (average r^2^ > 0.34) (Fig. 7). Similarly, three SNP loci (S3_1227787, S3_1227802, S3_1231197) formed a haplotype block with an average r^2^ > 0.64. Of the six significant SNPs associated with the *P. effusa* race 16 resistance, SNP S3_1050601 did not belong to any haplotype block. Haplotype alleles from block 1 and block 7 revealed a significant association with the *P. effusa* race 16 resistance (Table 2) and the haplotype GA, AT from block 1 and haplotype CGT, AAC in block 7 showed a large difference in frequency between resistant and susceptible groups.

**Table 2:** Haplotype association analysis at the *P. effusa* race 16 resistance locus in population segregating from cultivar Whale

SNP haplotypes^a^	Haplotypes		Ratios^b^		-log_10_P value^c^	Candidate genes^d^
	Alleles	Frequency	Susceptible	Resistant		
S3_658,306, S3_692697	GA	0.5	0.75	0.4	8.5	Spo12736, Spo12784
	AT	0.29	0.1	0.37	6.2	
S3_1227787, S3_1227802, S3_1231197	CGT	0.45	0.24	0.54	6.4	Spo12821
	AAC	0.39	0.67	0.28	11	

^a^ The SNPs are named for chromosome and position. Here S3_658,306 means SNP loci are located on chromosome 3 and positioned at 658306 bp

^b^ Ratio of haplotype alleles among the resistant and susceptible panels

^c^ Haplotype blocks were identified, and association analysis performed in Haploview 4.2 software [38]

^d^ Candidate genes were searched in the spinach genome [22] available at http://www.spianchbase.org. Disease resistance genes within the haplotype block or nearby the haplotype blocks that contain the associated SNPs were reported. The Spo12736 and the Spo12784 gene, both annotated as NB-ARC; leucine-rich repeat (LRR) is 8.92 Kb downstream and 2.69 Kb upstream of the *Pfs*16 associated SNP S3_658,306 and S3_692697. The second haplotype block does not harbor the disease resistance gene, but the SNP S3_122787 is only 7.86 Kb downstream of the Spo12821 gene annotated as CC-NBS-LRR disease resistance protein

Six high confidence SNPs were associated with the *P. effusa* race 16 resistance in this panel. The associated SNP regions were further explored to refine the downy mildew resistance candidate genes. This study maps the *RPF*3 locus in an interval of 0.57 Mb of chromosome 3 using the structured population of 172 lines. All six SNPs were mapped to the proximal end of chromosome 3, particularly in three physical regions: 0.66–0.69 Mb, 1.05 Mb, and 1.23 Mb (Fig. 7). These three regions harbor disease resistance candidate genes Spo12736, Spo12784, Spo12908, and Spo12821 (Table 1) within 2.69–11.28 Kb of the peak SNPs.

Five associated SNPs formed two haplotype blocks in Haploview analysis, and genes within or near the haplotype blocks increase our confidence in branding them as the candidate genes. Two disease resistance candidate genes were present within LD block 1. The Spo12736 gene is 8.92 Kb downstream from the SNP S3_658,306, while the Spo12784 gene was 2.69 Kb upstream of the SNP S3_692697. On the other hand, LD block 7 did not harbor any candidate disease resistance gene, but the associated SNP S3_122787 was only 7.86 Kb downstream of the Spo12821 gene. SNP S3_1050601 did not form a group but was in LD with the markers in block 7 (r^2^ ~ 0.34–0.49). The four candidate genes (Spo12736, Spo12784, Spo12908, and Spo12821) lying within or near the associated SNP or LD blocks were annotated as NB-ARC leucine-rich-repeat (LRR) disease resistance protein and CC-NBS-LRR disease resistance protein in the SpinachBase (http://www.spinachbase.org). All the identified candidate genes contain nucleotide-binding and leucine-rich-repeat domains that comprise most of the molecularly characterized resistance genes in plants. The proximal end of chromosome 3 harbors several other annotated disease resistance genes [22, 23], and the *RPF*1, *RPF*2, and *RPF*3 loci [19, 20, 24] were mapped in the same region (Fig. 7).

### Selection potential of associated SNP

For all SNPs associated with resistance against *P. effusa* race 16 from cultivar Whale, selection accuracy and efficiency were calculated. The selection accuracy of the associated SNPs varied from 83.9–92.7%, with an average of 86.0% (Table 3). The selection efficiency ranged from 46.3 to 61.5%, with an average of 55.7% (Table 3).

**Table 3:** Selection accuracy and efficiency of the significantly associated SNP markers for association panel of spinach population generated by crossing Whale and Lazio. The association panel comprises 172 spinach lines, of which 123 and 49 were resistant and susceptible to race 16 of the *P. effusa* race. For each SNP, the number of lines under each genotypic class was counted for the full panel, the resistant, and susceptible groups to calculate selection accuracy and efficiency

SNP marker	SNP type	Genotype	Number of lines and percentage						Selection Accuracy (%)^a^	Selection efficiency (%)^b^
			All (172)	Percent	Susceptible (49)	Percent	Resistant (123)	Percent		
S3_658,306	G/**A**	GG	56	39.2	30	71.4	26	25.7	86.2	52.4
		AG	41	28.7	7	16.7	34	33.7		
		AA	46	32.2	5	11.9	41	40.6		
		NN	29		7		22			
S3_692697	A/**T**	AA	82	50.0	42	87.5	40	34.5	92.7	46.3
		AT	58	35.4	2	4.2	56	48.3		
		TT	24	14.6	4	8.3	20	17.2		
		NN	8		1		7			
S3_1050601	**T**/A	TT	49	28.7	9	18.4	40	32.8	84.6	60.8
		AT	74	43.3	10	20.4	64	52.5		
		AA	48	28.1	30	61.2	18	14.8		
		NN	1		0		1			
S3_1227787	**C**/A	CC	58	38.2	9	20.0	49	45.8	84.3	56.6
		AC	44	28.9	7	15.6	37	34.6		
		AA	50	32.9	29	64.4	21	19.6		
		NN	20		4		16			
S3_1227802	**G**/A	GG	58	38.2	9	20.0	49	45.8	84.3	56.6
		AG	44	28.9	7	15.6	37	34.6		
		AA	50	32.9	29	64.4	21	19.6		
		NN	20		4		16			
S3_1231197	**T**/C	TT	58	34.3	7	14.3	51	42.5	83.9	61.5
		CT	66	39.1	13	26.5	53	44.2		
		CC	45	26.6	29	59.2	16	13.3		
		NN	3		0		3			

^a^ Selection accuracy (%) is the ratio of resistant lines with beneficial SNPs divided by the sum of resistant and susceptible lines with beneficial SNPs

^b^ Selection efficiency (%) is the ratio of resistant lines with beneficial SNPs divided by the total number of genotyped lines

**Fig. 8:**
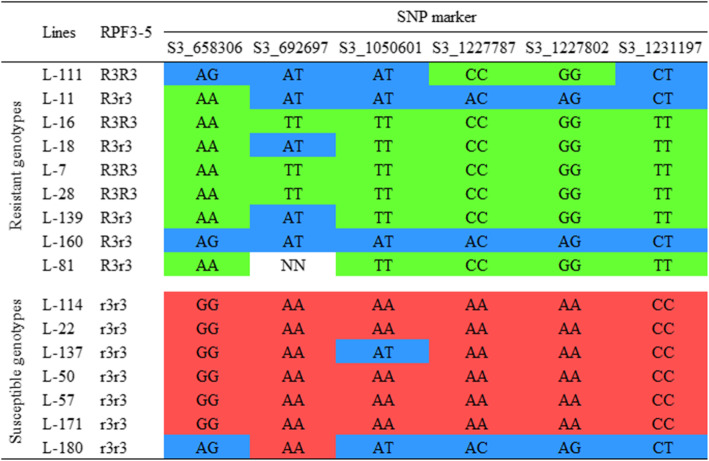
Visualization of nine resistant and seven susceptible genotypes at the significantly associated SNP loci. The SNP position of the associated SNP markers represents the physical location in chromosome 3 of the Sp75 spinach reference genome. A co-dominant marker RPF3–5 [19] cosegregating with the *RPF*3 locus was used to confirm resistance-susceptibility response in the seedling genotypes

A previously developed RPF3–5 marker linked to the *RPF*3 locus was assayed in 20 seedlings to investigate the predicted response and usefulness of the associated SNP marker in this study. The RPF3–5 marker correctly predicted disease response for 16 seedlings, while mismatches were found on the remaining four seedlings, showing 80% correspondence between phenotype score and marker prediction. The RPF3–5 marker indicated three susceptible seedlings as resistant and one resistant seedling as susceptible of the four mismatches. The downy mildew resistance-associated SNPs of the 16 seedlings were examined and observed in detail for their allelic distribution between the resistant and susceptible panels (Fig. 8). The graphic genotype panel (Fig. 8) shows three resistant plants were fixed for the resistant allele and the remaining resistant plants carry a single allele at all associated loci. Similarly, all susceptible plants except one were fixed for the susceptible associated allele. The resistant lines show a mixture of both resistant and susceptible alleles (36 fixed out of 54), while the susceptible plants were more fixed (36 out of 42) for susceptible alleles (Fig. 8).

## Discussion

### Downy mildew resistance

Downy mildew infestation reduces the yield and quality of fresh-market spinach as a low threshold (< 5%) of infected leaves makes the crop unmarketable and involves an additional labor cost to manually remove infested plants in the commercial field [19]. Utilization of genetic resistance offers an efficient disease control method and has been adopted to control downy mildew in spinach [15]. However, the rapid emergence of the new *P. effusa* races is continually overcoming the genetic resistance deployed in the newly released cultivars [15, 19]. Spinach breeding relies on the planned deployment of resistance genes from two parents in a hybrid cultivar. For any new *P. effusa* races, breeding involves screening the resistance sources to identify race-specific resistance loci to incorporate in the new cultivars [15]. The need for stable resistant cultivars against all known DM races is vital, and hence identification and mapping of downy mildew resistance loci from the cultivars, germplasm collections, and wild species are prioritized to combat rapidly evolving virulent races. Genetic characterization of the available resistance sources and the identification of tightly linked markers allow adopting a marker-based selection system to develop new cultivars. Traditional screening and selection methods based on evaluating the whole plants are labor-intensive, *P. effusa* being an obligate biotroph requires living tissue for sporangia production, and the downy mildew phenotyping requires environment-controlled facilities [12]. Identifying markers and adopting marker assisted selection (MAS) would expedite and ease the selection of downy mildew resistant spinach lines, and the marker based selection would be more efficient in terms of cost and resource needed. Thus, molecular markers are being developed in spinach [19–21, 24]. Characterizing each *RPF* locus and identifying gene-based markers will enhance the efficiency and precision of selection in developing new downy mildew resistance cultivars. Indeed identification of new resistance loci with a different mechanism and linked DNA markers will make the pyramiding or stacking of multiple resistance loci (*RPF*s) into a single cultivar feasible. Cultivars stacked with multiple resistant genes are attractive options for the spinach industry as they are considered to be durably resistant because the simultaneous evolution of new virulent races against multiple resistant genes is less likely to occur [39]. Current commercial spinach cultivars are hybrids combining multiple resistant loci effective against different sets of *P. effusa* races.

Following the downy mildew evaluation on a panel of seedling population segregating from cultivar Whale, around 71% of genotypes were identified as resistant to race 16 of *P. effusa*. Whale and Lazio are commercially available hybrid spinach cultivars containing different *RPF* genes, and around 60 plants of each cultivar were kept together in an isolation chamber and allowed to cross. As a result, phenotype and marker data did not fit a 1:1 ratio in this panel as expected for the regular pseudo-F2 population generated from a cross between expected heterozygous resistant (Rr) and non-resistant (rr) parents. In addition, the resistant F1 lines that were used for the cross might contain a mixture of Rr and rr plants as the plants were not tested with *P. effusa* or RPF markers. Spinach is commonly a dioecious crop having separate male and female plants, although some monoecious plants are found [18]. Both males and females were present in the crossing chamber, and thus selfing between male and female Lazio plants (Rr x Rr) might have occurred, resulting in inbreeds in the seeds lots, and some crosses may have been more successful than others leading to such segregation ratio. In this regard, association studies offer a good alternative as no biparental populations were required to map the locus; and thus, association analysis was pursued to map the resistance loci in this study.

### GWAS analysis for downy mildew resistance

Downy mildew disease response showed a bimodal distribution with 123 resistant and 49 susceptible genotypes. A major gene governs resistance to downy mildew in spinach. The resistant parent Whale carries the *RPF*3 locus and their response to available races of *P. effusa* have are known [5, 6]. Hence, association analysis was conducted using a binary or qualitative disease score to extend our understanding of the genes providing resistance at a higher resolution in spinach cultivar Whale. Principal components analysis was conducted to identify population structure and use the PCA covariates to correct the sub-population structure. The spinach association panel was subdivided into two subpopulations in both principal component analysis and the ADMIXTURE analysis. However, the resistance and susceptible genotypes were present in both groups, and no strong relationship between genetic structure and the resistance phenotype was observed (Fig. 3). PCA and relationship matrices were used as covariates in the mixed model analysis in TASSEL, PLINK, and GENESIS programs to account for the population structure and relatedness effect. The effect of population stratification was corrected using PCA covariates in all tested association models, as evidenced by QQ plots (Figs. 4, 5, 6). Furthermore, Bonferroni correction (LOD value > 5.34) was used to control the spurious association.

Association analysis was performed using multiple models and programs to sort consensus sets of SNPs and increase the confidence of the detected SNPs. The single marker regression model without any covariates identified the same set of markers as the general linear model that includes PCA covariates in TASSEL, which was slightly more than the mixed linear model in TASSEL that accounts for population structure and kinship (Table 1). The principal component covariates-based logistic regression model in PLINK detected only one SNP that passed the Bonferroni threshold. Similarly, GENESIS’s mixed linear model detected an additional three SNPs not identified in the TASSEL mixed model. In general, association analysis from multiple programs detected three hotspot regions on chromosome 3 associated with *P. effusa* race 16 resistance*.* The genomic position of the significant SNPs detected in multiple models was examined to identify nearby disease-resistant candidate genes. Downy mildew resistance gene in Whale maps to a 0.57 Mb interval containing four plant defense regulating genes in three nearby zones. Association results from this study falls in the same region as the previously mapped *RPF* genes in spinach [19, 23, 24], illustrating that the use of a small panel of breeding population (bi-parental, multi-parent, or mixed progenies) were efficient to identify the association with the qualitative traits and map the locus in spinach.

Selection accuracy and efficiency were calculated for significantly associated SNPs from multiple association models (Table 3). The selection accuracy and efficiency were medium to high (> 50%), and these markers were suitable to search for candidate genes. A very high selection efficiency is expected for the lines generated from a cross of two-parent cultivars; however, phenotyping errors from disease escape and contamination with multiple races impose a lower marker-trait association and selection efficiency. The phenotype score of seedlings and their corresponding RPF3–5 predicted phenotype score shows an 80% match indicating some inconsistencies between marker and disease scores in this population. Although uncommon in our experiments, such deviating disease response may have arisen from the disease escape, a condition where the susceptible plants do not show disease signs and symptoms. The other scenario may be the mixed pathogen races and isolates. Visualization of the SNP genotypic data among the selected resistant and susceptible progeny populations showed the susceptible panels were fixed for alleles compared to resistant panels in this study (Fig. 8) that may facilitate selection using associated markers. However, some of the alleles associated with the resistance are present in reference cultivar Sp75 [22], which does not contain the *RPF*3 locus. Therefore, the *RPF*3 associated SNPs reported here should be tested in broad genetic backgrounds to confirm their potential to differentiate the *RPF*3 locus before implementing for breeding and selection purposes. We have performed resequencing for 480 worldwide spinach germplasm accessions, including the Whale, Lazio, and around 40 commercial cultivars. The GWAS analysis in the diverse panel will provide more insights on alleles associated with *P. effusa*, and the *RPF*3 associated SNP markers identified in this study will be used to search for their potential association in the resequenced panel.

Resistance to downy mildew disease in spinach is hypothesized to be governed by a major gene with a substantial effect on phenotype. Despite expected high LOD and R^2^ values for the resistant locus, medium LOD and R^2^ values, on average of 5.34–9.6 and 20%, were observed for the SNP markers associated with the *Pfs* 16 resistance. The low LOD might be because the associated SNPs are far from the candidate genes, the population investigated here was developed from a cross of multiple male parent lines providing susceptible inbreeds, and the disease escapes. Spinach is an open-pollinated and highly heterozygous species, and the linkage disequilibrium decay is faster and is estimated at around 4 Kb in spinach [22]. On the other hand, multiple minor effect genes or a gene with multiple alleles might control the resistance, so the associated SNP region has shown lower values of association (LOD values). Despite the moderate LOD and R^2^ values observed, the current result provided a high-resolution characterization of the *RPF*3 resistance locus. Additional information from this report and further understanding of the genetic mechanism underlying the resistance may help downy mildew resistance breeding and deploy the resistance alleles.

### Candidate genes associated with *P. effusa* race 16

Polymorphisms in the causal genes regulating the phenotypic differences are of biological interest. Identification of candidate genes aids in functional characterization and identification of polymorphisms within the functional genes. Common SNPs identified from multiple association models were pursued to search for disease resistance candidate genes. None of the associated SNPs fell on the gene region responsible for disease resistance; however, most of the associated SNPs were within 10 Kb of the gene with functions pertinent to disease resistance. SNPs S3_658,306 and S3_692697 associated with *Pfs* 16 resistance in this study were in an LD block and harbored the two disease resistance genes Spo12736 and Spo12784. Another SNP S3_1050601 associated with *P. effusa* race 16 resistance was close to disease resistance gene Spo12908, but this SNP was not in LD with other nearby SNPs. Similarly, three SNPs (S3_1227787, S3_1227802, S3_1231197) in an LD block were less than 8 Kb from the disease resistance gene Spo12821.

The proximal end of chromosome 3 contains 14 annotated disease resistance genes [22–24], and the markers for *RPF*1, *RPF*2, and *RPF*3 were mapped in the same region [19–24]. The *RPF*1 locus was narrowed to a 1.5 Mb [19], 0.89 Mb [23], and 0.84 Mb region [24]. Based on the NBS-LRR domain in the spinach genome, five genes (Spo12736, Spo12784, Spo12903, Spo12905, and Spo12821) were predicted as potential downy mildew resistance candidate genes [22]. Recently, amino acid conserved domain analysis between the *RPF*1 resistant and susceptible lines identified Spo12729, Spo12784, and Spo12903 as the candidate genes [23]. And following the association analysis performed in the segregating population generated from a cross of multiple resistant parents reported Spo12784, Spo12903, Spo12905, and Spo12821 as the potential candidate genes involved in providing resistance against the downy mildew pathogen [24].

The *RPF*3 locus was characterized in this study using association analysis in the breeding population derived from cultivar Whale. The resistance locus was mapped to the three genomic regions (0.66–0.69 Mb, 1.05 Mb, 1.23 Mb) of chromosome 3. Four genes (Spo12736, Spo12784, Spo12908, and Spo12821) in the vicinity of peak SNPs were identified as the most probable candidate genes. The candidate genes were annotated as NB-ARC leucine-rich-repeat (LRR) and CC-NBS-LRR disease resistance protein (Table 1). The NBS-LRR domains are the most common plant disease resistance genes acting as a receptor of pathogen effectors and activating the signaling cascades for defense [40]. *RPF*3 gene postulated from the current mapping effort falls in the same region as reported in earlier work, but the region (0.66–1.23 Mb) contains more than ten disease resistance genes [22, 23]. Three of the four candidate genes identified in this work except Spo12908 were reported as downy mildew resistance candidate genes in [22]. Candidate gene Spo12784 identified for the *RPF*3 locus in this study was also reported as a candidate gene for the *RPF*1 locus by She et al. [23]. Candidate genes Spo12784 and Spo12821 identified here were also reported in the study of Bhattarai et al. [24].

The *RPF* locus (*RPF*1 through *RPF*6) has been established in spinach, and these loci are being characterized at the genetic level [19, 24]. Effort and emphasis have been proposed to clone the *RPF*1 gene and validate the functions in disease resistance. Additional characterization and discovering major and minor downy mildew resistance genes are essential as the downy mildew pathogen with a high potential to evolve with new virulences may quickly overcome the known resistances deployed in the commercial cultivars. Detailed genetic characterization of the resistance genes and identification of breeder-friendly diagnostic markers will enable an increased selection efficiency to introgress resistant alleles in cultivar development. In addition, functional characterization of the R genes will explain the genetic and regulatory mechanism of host-pathogen interaction, disease development, mechanism of evolution of the new virulent races, and their strategy to break down the R genes. Such information on host-pathogen interactions may help formulate an improved strategic approach in spinach breeding and cultivar development.

Identification and development of functional markers residing on the gene are most desirable, but it warrants identification and cloning of genes with explained functions of the domains towards resistance-susceptibility. Alternatively, genetically linked and associated markers to the traits are commonly used in plant breeding programs to select plants with expected phenotypes based on the marker genotype data. And a large number of SNPs [41] and SSR markers [42] are available in spinach. With recent advancements in sequencing platforms and continuously reducing sequencing costs, the genome or transcriptome of large plant panels can be sequenced at a lower price. Whole-genome resequencing of spinach core collections has been recently completed, and the sequence-based genomic resources and millions of SNP of the core collections are available. The new sequence resources are expected to expand our current understanding of genetics, genomics, and biology of commercially important traits, including the resistance to downy mildew pathogen. In recent years, field trials were performed to evaluate USDA spinach core collections for tolerance to downy mildew in the commercial growing regions under natural inoculum pressure [27]. The GWAS analysis performed with the field tolerance data identified several associated SNP regions that could breed downy mildew tolerant lines [27, 43]. The qualitative and quantitative screening and mapping efforts are aimed to identify the diverse genetic mechanism and desirable alleles contributing to resistance and use them in pyramiding the race-specific major genes and minor genes in a single cultivar.

A GWAS analysis was performed in a set of 172 spinach genotypes and mapped a major locus resistant to race 16 of *P. effusa* to a 0.57 Mb interval of chromosome 3, and identified a set of SNP markers statistically associated with the resistance to *P. effusa*. The SNP loci are close to the candidate genes that govern disease resistance. The beneficial allele can be used in spinach breeding programs to select the resistance genotypes through MAS approaches. The set of SNP markers identified in this study and others identified from several ongoing studies will be re-tested and validated in multiple populations to extend their use as a KASP marker for their potential use in MAS and narrow down the downy mildew resistance *RPF*3 and other *RPF* locus.

Furthermore, validation of candidate genes Spo12736, Spo12784, Spo12908, and Spo12821 via gene-knockout and gene-expression experiments may confirm their involvement in providing resistance to downy mildew and explaining the molecular mechanism of resistance. Research and investigations are ongoing to expand the current understanding of host-pathogen interaction in spinach downy mildew, including identifying and mapping multiple resistance sources, a functional test of the *RPF* genes, and characterizing functions of the effector genes. From the perspective of rapidly emerging races that are breaking down the resistance deployed in commercial cultivars, the host-pathogen battle in spinach downy mildew system offers a model to understand and explore the continued host-pathogen win-lose interaction, and a newer understanding may help in formulating and adopting an improved downy mildew resistance breeding strategy. Future reports on an expanded knowledge of spinach-downy mildew host-pathogen interaction and functional characterization of genetic resistance will be of high value to the scientific community and implement genetic resistance against the downy mildew.

## Conclusions

The current study employing the association mapping approach identified downy mildew resistance loci in a population segregating from cultivar Whale. Six significant SNPs associated with *P. effusa* race 16 in this study were close to the annotated disease resistance genes. Candidate genomic regions associated with the *P. effusa* race 16 resistance and continual development of race-specific resistance markers will enhance the efficiency and precision of breeding downy mildew resistance cultivars. Indeed mapping and identifying new resistance loci and linked DNA markers may make the pyramiding or stacking of multiple *RPF* loci and minor QTLs into a single cultivar feasible. A spinach cultivar containing *RPF*1, *RPF*2, and *RPF*3 genes can tolerate *P. effusa* races 1 through 16, although the three *RPF* genes have not yet been stacked into a single cultivar using the regular breeding introgression procedure as the three genes are more likely the alleles of the single locus or are very closely linked, making the stacking of the resistance loci impractical. The development of gene-specific markers may help breeders achieve the goal of incorporating all genes in a single line. A single cultivar with multiple resistant genes is an attractive option for the spinach industry as the cultivars will be resistant to several pathogen races and may hinder the new evolving race from overcoming the known resistance genes.

## Methods

### Plant materials

Breeding population developed from a cross of cultivars Whale and Lazio was screened for resistance to the race 16 of *P. effusa* (isolate UA201519B) in this study. Whale contained the *RPF*3 locus and was resistant to *P. effusa* race 16, while the Lazio containing the *RPF*2 and *RPF*4 loci was susceptible [5, 6]. These cultivars are included in a set of differential cultivars to evaluate and compare downy mildew disease reactions. Seeds were obtained from Gowan Seed Company, Chualar, CA, and were grown at the USDA Crop Improvement and Protection Research Unit in Salinas, CA. Three-week-old Whale F1 and Lazio F1 plants (about 60 plants each) were moved to an isolation chamber in two rows for each cultivar and allowed to cross. Seeds were harvested from each female plant and bulked to represent the mixed population used in this study. Seeds were planted in 25 × 50-cm plastic trays filled with potting soil (Sun Gro Horticulture, Canada) at the University of Arkansas, Fayetteville, AR. Each plant tray contains ten rows, and 10–15 seeds/row was planted. Around 6–8 plants/ row were kept after germination and labeled, and plants were grown in the greenhouse (25 °C) for two weeks, watered daily, and fertilized weekly using Miracle-Gro® All Purpose Plant Food. The universal susceptible check cultivar Viroflay, and the two parents, Lazio, and Whale were also included as controls for phenotyping assay.

### Inoculation and phenotyping

A leaf was excised and stored for DNA extraction from each labeled seedlings before inoculation. The remaining plants were inoculated following the routine whole plant inoculation method [5, 6, 12, 24]. Briefly, the inoculum was increased on a susceptible cultivar Viroflay every week, and the fresh inoculum was used to inoculate the spinach population. Sporangia were washed off from the infected leaves of Viroflay in cold (4 °C) distilled water. Inoculum suspension was filtered using two layers of cheesecloth, diluted to 10^5^ spores/ml, and sprayed with a Badger basic spray gun (model 250) until the leaves were wet. Inoculated seedlings in trays were incubated in a dew chamber (18 °C) for 24 h. Following the dew chamber incubation, the plant trays were moved to a growth chamber (18 °C, 12 h dark-light cycle). After 6 days, plant trays were returned to the dew chamber (18 °C) for 24 h to induce sporulation, and disease reactions of each plant were scored.

Downy mildew disease reactions were scored qualitatively for incidence by visual inspection for the presence or absence of sporulation. Severity was scored quantitatively on a 0–100% scale, representing the percentage of leaf area covered with sporulation. Qualitative disease response was used as phenotype data to conduct association analysis.

### Sequencing and genotyping

Genotyping-by-sequencing (GBS) [44] was pursued to sequence the population and to identify Single Nucleotide Polymorphism (SNP) markers. Young leaves of each seedling were harvested before inoculation and stored at − 80 °C. Genomic DNA was extracted using the CTAB (cetyl trimethylammonium bromide) method. The DNA quality was checked on 1% agarose gel and quantified using NanoDrop. DNA was submitted for sequencing at the UW-Madison Biotech center, where DNA quality and integrity were re-checked using Quant-IT PicoGreen fluorescent dye (Thermo Fisher, Waltham, MA, USA). DNA was digested using *Ape*KI restriction enzyme, and digested fragments of each sample were ligated with unique barcode adapters and Illumina adapters. For GBS library preparations, samples were pooled in equal proportion and were amplified, purified, and sequenced as 150 bp paired-end reads on NovaSeq (Illumina, San Diego, CA, USA).

The raw sequence reads were preprocessed to remove sequencing adapters and filtered for low-quality bases for a minimum quality of Q20 using skewer [45]. The remaining good-quality reads were demultiplexed and aligned to the six chromosomal scaffolds of the spinach reference genome [22, 37] available at (http://spinachbase.org/) using Bowtie 2 software [46]. The aligned sequence reads were then analyzed with the TASSEL GBS version 2 pipeline [47, 48] for genotyping and SNP calling. VCFtools [49] was used to filter for multiallelic SNPs and to keep biallelic SNPs. Furthermore, SNPs were filtered in PLINK v1.9 [50, 51] for missing data (< 25%), individual missing (< 25%), minor allele frequency (MAF < 0.02), and Hardy Weinberg equilibrium (1e^− 07^) in both case and control. The distribution of filtered SNPs over the six chromosomes was presented by drawing a density plot using the CMplot package in R.

### Population structure analysis

Genetic structure was analyzed using a model-based clustering algorithm in ADMIXTURE v1.22 [52] to define the spinach panels subpopulation structure and assign each genotype to sub-population groups. The filtered SNP dataset was then pruned for linkage disequilibrium (LD) in PLINK (−-indep-pairwise 50 5 0.2 option) and correlated pairs of SNPs were removed. ADMIXTURE analysis was run with ten-fold cross-validation for one to ten groups on the LD pruned SNPs, and subpopulation group numbers were determined based on the lowest cross-validation error. Membership probability (Q matrices) estimates from ADMIXTURE were used to draw a barplot to visualize clustering among spinach genotypes. A cutoff probability value with Q > 0.75 was used to assign genotype to a cluster, while the genotypes with Q < 0.75 were kept in an admixed group.

Principal component analysis (PCA) was run in PLINK using the identity by state (IBS) matrix calculated with the LD pruned SNPs. The total number of principal components (PC) was chosen according to ADMIXTURE analyses and the PC matrices were used as a covariate in PLINK to control for population structure, and the PCA plot was drawn using the R package ggplot2.

### Genomewide association analysis

GWAS was conducted using the single marker regression (SMR) without controlling for structure and admixture, general linear model (GLM) by including PCA matrices, and the mixed linear model (MLM) using kinship matrix (K) and PCA matrices in TASSEL 5.2.31 [47]. The phenotype score was changed to 1 for resistant and 9 for susceptible disease response. MLM is widely used to analyze quantitative traits that assume errors are normally distributed, mutually independent, and are homoscedastic. However, fitting the MLM on the binary trait violates statistical assumptions and often results in an increased false-positive discovery rate, and hence association analysis was conducted on multiple programs, including models developed to analyze qualitative traits.

The GWAS signals identified in the TASSEL programs were further confirmed using the logistic regression model in the PLINK v1.9 and the logistic mixed model (LMM) in the GENESIS R Bioconductor package. Disease incidence data (0 = Resistant, 1 = Susceptible) were used as a binary phenotype trait to map the resistance loci in the PLINK and GENESIS model. Association analysis was performed in PLINK, including IBS distance-based PCA clusters as a covariate to control genetic relatedness.

GENESIS program uses mixed models to test genetic association using PC-AiR to compute principal components and use as fixed effect covariates to account for unknown and known relatedness between genotypes [53], and a kinship matrix (or genetic relationship matrix) estimated from PC-Relate to use as a random effect to account for phenotype correlation due to genetic similarity among samples [54]. The LD pruned SNPs were used to calculate the principal component and kinship matrix in GENESIS. Kinship matrix was added as a random effect in the null model, and the principal components were added as a fixed-effects covariate in the GWAS model to estimate the SNP association using the score test. Manhattan plots and QQplots from all models were generated using the qqman and CMplot package in R. Bonferroni correction (0.05/n) was used as a threshold for significance of marker-trait association and -log_10_(*P*) > 5.34 have been reported.

The final sets of SNPs identified from multiple GWAS models were compared for the significant difference in the two alleles for phenotypic values using *t*-test in an Excel spreadsheet.

### Haplotype analysis and candidate gene search

Haplotype analysis was performed in the *P. effusa* resistance associated region (0.60–1.40 Mb) of chromosome 3 in the Haploview 4.2 software [54] and the LD blocks were determined using the solid spline to LD method. For the same set of SNPs, the association of the haplotypes with the *P. effusa* race16 resistance was inferred in Haploview using a chi-square haplotype-test and a difference in haplotype frequencies between the resistant and susceptible panels was noted.

Highly significant SNPs identified from multiple association models were used to search for candidate genes in the spinach genome sequences [22, 37]. Initially, genes located within 12 Kb upstream and downstream of the peak SNPs in the spinach genome were searched. Genes providing disease resistances against plant pathogens were of interest and annotated functions of the potential candidate genes were reported. Furthermore, the haplotype blocks that contained the associated SNPs were searched for candidate genes. And for the blocks that harbored the associated SNPs but do not include disease resistance genes, the nearby genes within 10 Kb were reported as potential candidate genes.

### Selection accuracy and efficiency of associated SNP

Selection accuracy was calculated for all significantly associated SNP markers. For this, the total number of lines in each SNP genotype class and the number of resistant and susceptible lines per genotype class were counted. The selection accuracy and selection efficiency were calculated as described in [55] using the number of lines belonging to the respective genotype class for each SNP loci and as shown here:

$$ \mathrm{Selection}\ \mathrm{accuracy}\ \left(\%\right)=100\ \mathrm{x}\ \left[\mathrm{number}\ \mathrm{of}\ \mathrm{resistant}\ \mathrm{line}\mathrm{s}\ \mathrm{in}\ \mathrm{the}\ \mathrm{benefit}\ \mathrm{SNP}\ \mathrm{allele}/\left(\mathrm{number}\ \mathrm{of}\ \mathrm{resistant}\ \mathrm{line}\ \mathrm{in}\ \mathrm{the}\ \mathrm{benefit}\ \mathrm{SNP}\ \mathrm{allele}+\mathrm{number}\ \mathrm{of}\ \mathrm{susceptible}\ \mathrm{line}\ \mathrm{in}\ \mathrm{the}\ \mathrm{benefit}\ \mathrm{SNP}\ \mathrm{allele}\right)\right]. $$

$$ \mathrm{Selection}\ \mathrm{efficiency}\ \left(\%\right)=100\ \mathrm{x}\ \left(\mathrm{number}\ \mathrm{of}\ \mathrm{resistant}\ \mathrm{lines}\ \mathrm{in}\ \mathrm{the}\ \mathrm{benefit}\ \mathrm{SNP}\ \mathrm{allele}/\mathrm{total}\ \mathrm{number}\ \mathrm{of}\ \mathrm{genotyped}\ \mathrm{lines}\right). $$

Furthermore, the RPF3–5 marker linked to the *RPF*3 locus [19] was used in a blind test to screen 20 seedlings. The marker prediction and the disease scores were compared. Next, the downy mildew disease response scores of the seedlings, disease response predicted with RPF3–5 marker, and the SNPs genotype at the associated marker loci was visualized.

## Data Availability

The datasets generated and/or analyzed during this study are available in the FigShare repository, 10.6084/m9.figshare.14714352.v1 (Accession number: 14,714,352).

## Abbreviations

GBS: Genotyping by sequencing
DM: Downy mildew
RGA: Resistance gene analogs
SNP: Single nucleotide polymorphism
LD: Linkage disequilibrium
LOD: Logarithm of odds
